# Prevalence, awareness, treatment, and control of hypertension based on ACC/AHA versus JNC7 guidelines in the PERSIAN cohort study

**DOI:** 10.1038/s41598-022-07896-9

**Published:** 2022-03-08

**Authors:** Sadaf Sepanlou, Farid Najafi, Hossein Poustchi, Mahboubeh Parsaeian, Ali Ahmadi, Mohammadhossein Somi, Farhad Moradpour, Reza Alizadeh-Navaei, Ali Gohari, Bijan Zamani, Ali Esmaeilinadimi, Abbas Rezaianzadeh, Fariborz Mansour-Ghanaei, Ehsan Bahramali, Alireza Ansari-Moghaddam, Behrooz Hamzeh, Elham Zanganeh Yousefabadi, Mohammad Javad Zare Sakhvidi, Iraj Mohebbi, Mohammad Reza Fattahi, Azim Nejatizadeh, Hossein Marioryad, Nazgol Motamed-Gorji, Farzin Roozafzai, Sareh Eghtesad, Zahra Mohammadi, Amaneh Shayanrad, Maryam Sharafkhah, Arash Etemadi, Farin Kamangar, Stephen P. Juraschek, Reza Malekzadeh

**Affiliations:** 1 Digestive Disease Research Center, Digestive Diseases Research Institute, Tehran University of Medical Sciences, Tehran, Iran; 2 Research Center for Environmental Determinants of Health, Health Institute, Kermanshah University of Medical Sciences, Kermanshah, Iran; 3 Liver and Pancreatobiliary Diseases Research Center, Digestive Diseases Research Institute, Tehran University of Medical Sciences, Tehran, Iran; 4 Department of Epidemiology and Biostatistics, School of Public Health, Tehran University of Medical Sciences, Tehran, Iran; 5 Department of Epidemiology and Biostatistics, Modeling in Health Research Center, School of Health, Shahrekord University of Medical Sciences, Shahrekord, Iran; 6 Liver and Gastrointestinal Diseases Research Center, Tabriz University of Medical Sciences, Tabriz, Iran; 7 Social Determinants of Health Research Center, Research Institute for Health Development, Kurdistan University of Medical Sciences, Sanandaj, Iran; 8 Gastrointestinal Cancer Research Center, Non-Communicable Diseases Institute, Mazandaran University of Medical Sciences, Sari, Iran; 9 Non-Communicable Diseases Research Center, Sabzevar University of Medical Sciences, Sabzevar, Iran; 10 Digestive Disease Research Center, Ardabil University of Medical Sciences, Ardabil, Iran; 11 Non-Communicable Diseases Research Center, Rafsanjan University of Medical Sciences, Rafsanjan, Iran; 12 Colorectal Research Center, Shiraz University of Medical Sciences, Shiraz, Iran; 13 Gastrointestinal and Liver Diseases Research Center, Guilan University of Medical Sciences, Rasht, Iran; 14 Noncommunicable Diseases Research Center, Fasa University of Medical Sciences, Fasa, Iran; 15 Health Promotion Research Center, Zahedan University of Medical Sciences, Zahedan, Iran; 16 Department of Internal Medicine, Imam Khomeini Hospital, Ahvaz Jundishapur University of Medical Sciences, Ahvaz, Iran; 17 Occupational Health Research Centre, School of Public Health, Shahid Sadoughi University of Medical Sciences, Yazd, Iran; 18 Social Determinants of Health Research Center, Clinical Research Institute, Urmia University of Medical Sciences, Urmia, Iran; 19 Gastroenterohepatology Research Center, Shiraz University of Medical Sciences, Shiraz, Iran; 20 Molecular Medicine Research Center, Hormozgan University of Medical Sciences, Bandar Abbas, Iran; 21 Yasuj University of Medical Sciences, Yasuj, Iran; 22 Division of Cancer Epidemiology and Genetics, National Cancer Institute, Bethesda, MD USA; 23 Department of Biology, School of Computer, Mathematical, and Natural Sciences, Morgan State University, Baltimore, MD USA; 24 Department of Medicine, Beth Israel Deaconess Medical Center, Harvard Medical School, Boston, MA USA; 25 Digestive Oncology Research Center, Digestive Diseases Research Institute, Shariati Hospital, Tehran University of Medical Sciences, North Kargar Ave., Tehran, 14117-13135 Iran

**Keywords:** Cardiology, Diseases, Medical research, Epidemiology, Risk factors

## Abstract

In this cross-sectional population-based study, we used the baseline data of the Prospective Epidemiologic Research Studies in IrAN cohort study collected in Iran from 2014 to 2020. The main outcomes were the prevalence of hypertension and proportion of awareness, treatment, and control based on the 2017 ACC/AHA guideline compared to the seventh report of the Joint National Committee (JNC7). Of the total of 163,770 participants, aged 35–70 years, 55.2% were female. The sex-age standardized prevalence of hypertension was 22.3% (95% CI 20.6, 24.1) based on the JNC7 guideline and 36.5% (31.1, 41.8) based on the ACC/AHA guideline. A total of 24,312 participants [14.1% (10.1, 18.1)] were newly diagnosed based on the ACC/AHA guideline. Compared to adults diagnosed with hypertension based on the JNC7 guideline, the newly diagnosed participants were mainly young literate males who had low levels of risk factors and were free from conventional comorbidities of hypertension. About 30.7% (25.9, 35.4) of them (4.3% of the entire population) were eligible for pharmacologic intervention based on the ACC/AHA guideline. Implementation of the new guideline may impose additional burden on health systems. However, early detection and management of elevated blood pressure may reduce the ultimate burden of hypertension in Iran.

## Introduction

In 2017, the American College of Cardiology/American Heart Association (ACC/AHA) guideline was released^1^, in which lower thresholds (≥ 130/80 mmHg) were recommended for hypertension, and the upper end of prehypertension based on the seventh report of the Joint National Committee (JNC7)^2,3^ was reclassified as stage 1 hypertension. The rationale for this shift is the evidence showing that adults with blood pressure in this range have an approximately twofold increase in risk of cardiovascular diseases (CVDs) compared to adults with normal blood pressure^4,5^. Additionally, recent randomized clinical trials have demonstrated benefits from a systolic blood pressure (SBP) lower than 130 mm Hg^6,7^ including the Systolic Blood Pressure Intervention Trial (SPRINT) which demonstrated substantial reduction in CVD events by applying an intensive systolic blood pressure target < 120 mmHg^8^. In a very recent study, Whelton et al.^9^ reported that the stepwise rise in incident atherosclerotic CVDs and presence of coronary artery calcium begins at SBP levels as low as 90 mmHg. However, the implications of the new hypertension definitions are under debate. Using lower thresholds for definition of hypertension will lead to increase in estimated prevalence, which will impose additional burden on health systems especially in low-middle income countries with limited resources^10–13^. On the other hand, early diagnosis and treatment of high blood pressure among adults previously classified in the category of “pre-hypertension” may lead to reduced all-cause and CVD-specific mortality and morbidity^5,13^. Primordial prevention seems to be a necessity for maintaining optimal blood pressure levels even in adults free from traditional risk factors of CVD^9^.

Studies demonstrate that all-cause mortality and cardiovascular deaths attributable to high blood pressure doubled in Iran since 1990 and hypertension is the most important risk factor responsible for mortality in both sexes^14,15^. JNC7 is still the widely used guideline for definition and treatment of hypertension in Iran, specifically among general physicians in remote areas. However, recent guidelines are gradually becoming popular. It is of utmost importance to explore the impacts of stricter definitions for high blood pressure prevalence, treatment, and control to reduce the burden of CVD in a country with a high prevalence of hypertension as a middle-income nation. There is a slowly increasing trend towards using ACC/AHA guideline in Iran during the past couple of years. Therefore, exploring the potential impact of this guideline on definition, treatment, and control of hypertension in Iran is essential.

The main objective of the current study was to determine the impact of the two guidelines on estimated prevalence, awareness, treatment, and control of hypertension among a very large group of Iranians residing in various regions across the country.

## Results

A total of 163,770 participants were recruited from 2014 to 2020, 115,979 (70.8%) participants lived in urban areas and 47,791 (29.2%) participants were rural dwellers. A total of 90,397 participants (55.2%) were female. The mean (SD) age of the participants was 49.4 (9.2) years and 35.5% of participants were in the 35–44 age category. A total of 33,675 participants (20.6%) had no schooling. Systolic and/or diastolic blood pressure was missing in 889 participants.

**Figure 1:**
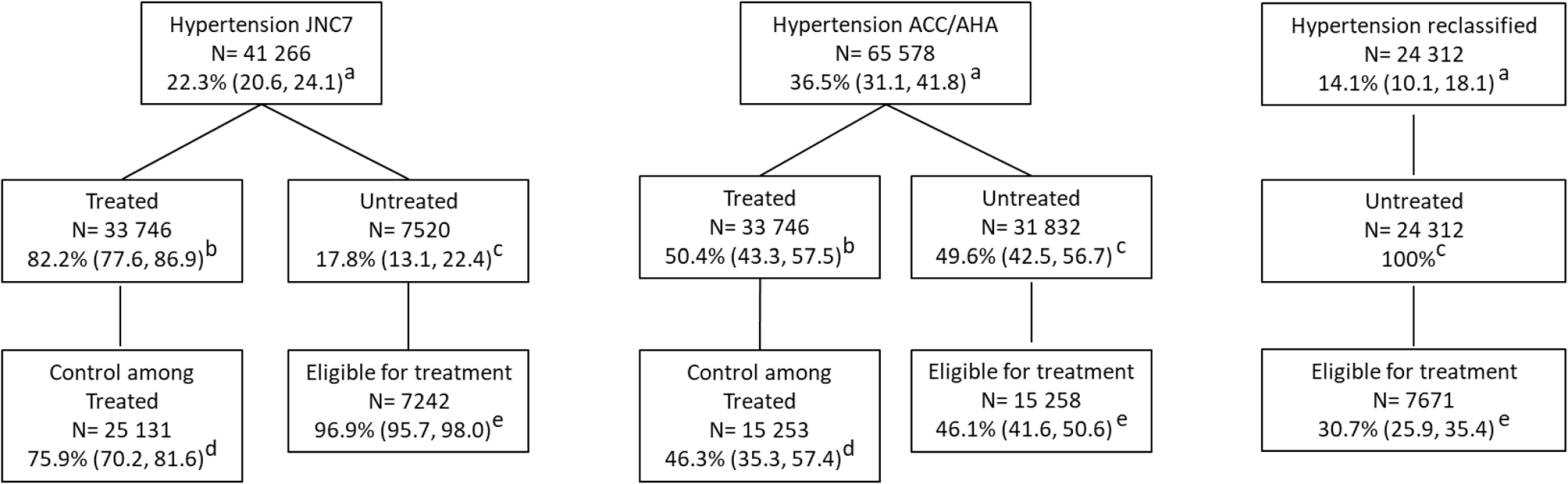
Number and weighted prevalence of hypertension, and proportion of treatment, control, and eligibility for pharmacologic intervention among adults classified as hypertensive based on both guidelines and the group of adults reclassified based on ACC/AHA guideline. ^a^Prevalence of hypertensive participants among all study population. ^b^Proportion of hypertensive participants who are treated. ^c^Proportion of hypertensive participants who are untreated. ^d^Proportion of control among treated hypertensive participants. ^e^Proportion of untreated hypertensive participants eligible for pharmacologic intervention.

**Figure 2:**
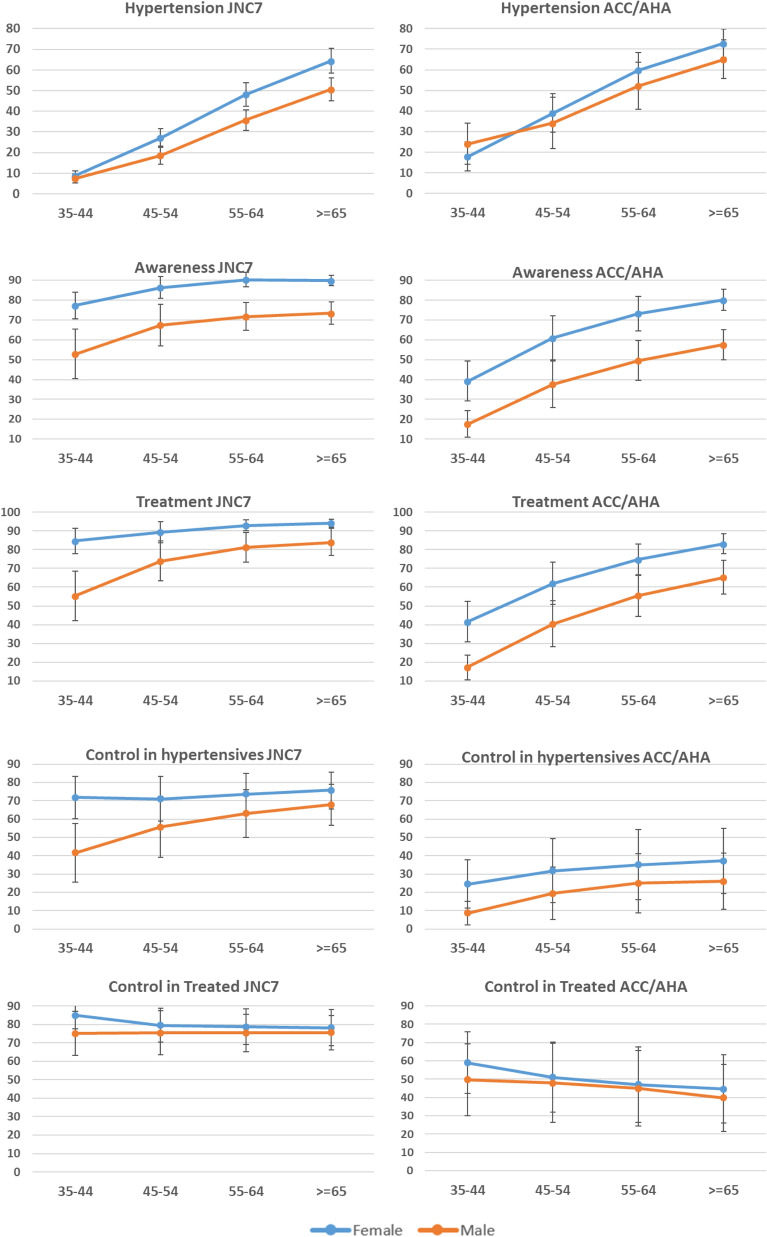
Sex and age-specific prevalence of hypertension and proportion of awareness, treatment, and control based on the two guidelines.

The sex-age standardized prevalence of hypertension was 22.3% (95% CI 20.6, 24.1) based on the JNC7 guideline and 36.5% (31.1, 41.8) based on the 2017 ACC/AHA guideline, which was 14.1% higher in absolute terms and 63.7% higher in relative terms (Fig. 1). The prevalence of hypertension based on the JNC7 guideline was significantly higher among females compared to males. In contrast, there was no difference in hypertension prevalence between sexes based on the ACC/AHA guideline. The prevalence of hypertension was greater with age regardless of guideline (Fig. 2).

A total of 24,312 (14.1% [10.1, 18.1]) participants who were classified in the category of “pre-hypertension” were reclassified into the hypertension category based on the ACC/AHA guideline (Fig. 1). Compared to adults traditionally diagnosed with hypertension based on the JNC7 report, the newly-diagnosed hypertensive adults based on the ACC/AHA guideline were more commonly male (62.1 vs. 42.9%), were younger than 55 years old (75.8 vs. 46.7%), were literate (74.5 vs. 57.4%), had normal BMI (23.2 vs. 15.5%), had high physical activity (39 vs. 29.5%), were free from diabetes (87.1, vs. 68.1%), were free from CKD (86.6 vs. 70.6%), had normal serum lipids (62.3 vs. 50.4%) and had low 10 year risk of ASCVD (90.1 vs. 72.5%) (Table 1). The shift from prehypertension based on JNC7 to stage 1 hypertension based on the ACC/AHA guideline was more prominent in males (Table 2).

**Table 1:** Weighted prevalence of hypertension based on the 2017 ACC/AHA and JNC7 hypertension guidelines across socio-demographic groups, and increase in prevalence defined based on the ACC/AHA guideline.

	JNC7 (N = 41,266)	ACC/AHA (N = 65,578)	Reclassified participants (N = 24,312)	Relative Difference in prevalence (%)
**Sex**				
Male	18.9 (16.9, 20.9)	36.1 (29.6, 42.6)	17.2 (12.4, 22.1)	91
Female	25.9 (24.2, 27.7)	36.8 (32.3, 41.4)	10.9 (7.6, 14.1)	42.1
**Age categories**				
35–44	8.0 (6.7, 9.2)	21.9 (16.4, 27.5)	14.0 (9.6, 18.4)	173.8
45–54	22.9 (20.4, 25.4)	38.1 (31.8, 44.3)	15.2 (10.9, 19.4)	66.4
55–64	41.9 (39.2, 44.6)	55.9 (50.6, 61.1)	14.0 (10.4, 17.6)	33.4
≥ 65	57.9 (54.8, 60.9)	68.4 (64.2, 72.6)	10.5 (8.1, 13.0)	18.1
**Residence**				
Urban	22.5 (20.5, 24.5)	35.5 (29.0, 42.0)	13.0 (8.2, 17.8)	57.8
Rural	21.9 (18.2, 25.6)	39.8 (33.5, 46.1)	17.9 (15.1, 20.7)	81.7
**Marital status**				
Non-married	31.5 (28.6, 34.4)	43.2 (38.1, 48.2)	11.7 (8.2, 15.1)	37.1
Married	21.6 (19.8, 23.3)	35.9 (30.5, 41.4)	14.3 (10.3, 18.4)	66.2
**Education**				
Illiterate (no schooling)	36.0 (32.2, 39.8)	49.6 (44.2, 55.1)	13.6 (10.1, 17.2)	37.8
≤ 5 years (primary)	21.4 (18.8, 24.1)	35.8 (30.9, 40.7)	14.4 (10.6, 18.2)	67.3
6–8 years (middle)	15.3 (13.1, 17.4)	30.0 (23.6, 36.4)	14.7 (10.0, 19.5)	96.1
9–12 years (secondary)	15.7 (13.7, 17.7)	29.5 (23.7, 35.4)	13.8 (9.7, 18.0)	87.9
> 12 years (university)	15.5 (13.5, 17.5)	29.8 (23.0, 36.5)	14.3 (9.2, 19.3)	92.3
**Wealth index**				
Quintile 1 (poorest)	27.8 (24.1, 31.6)	42.8 (38.4, 47.1)	14.9 (11.6, 18.3)	54
Quintile 2	24.5 (21.9, 27.0)	39.2 (35.1, 43.2)	14.7 (11.2, 18.2)	60
Quintile 3	22.5 (20.5, 24.4)	36.4 (30.9, 41.8)	13.9 (9.9, 17.9)	61.8
Quintile 4	19.5 (17.4, 21.7)	33.3 (27.1, 39.6)	13.8 (9.4, 18.2)	70.8
Quintile 5 (richest)	19.5 (17.1, 21.9)	33.0 (25.6, 40.4)	13.5 (8.2, 18.8)	69.2
**Body Mass Index (kg/m**^**2**^**)**				
Underweight	5.1 (4.1, 6.2)	16.0 (11.3, 20.8)	10.9 (6.5, 15.2)	213.7
Normal	13.2 (11.4, 14.9)	25.6 (20.8, 30.5)	12.5 (9.0, 15.9)	93.9
Overweight	21.8 (19.3, 24.4)	36.4 (29.7, 43.1)	14.5 (10.1, 18.9)	67
Obese	31.9 (29.1, 34.8)	47.1 (39.8, 54.4)	15.2 (10.4, 19.9)	47.6
**Physical activity**				
Low activity	27.8 (25.6, 29.9)	42.2 (36.3, 48.1)	14.5 (9.9, 19.0)	51.8
Medium activity	22.8 (21.2, 24.4)	36.0 (30.6, 41.4)	13.2 (9.0, 17.3)	57.9
High activity	17.6 (16.0, 19.2)	32.2 (27.4, 37.0)	14.6 (11.0, 18.2)	83
**Waist to hip ratio**				
Normal	9.6 (8.3, 10.9)	22.0 (18.2, 25.9)	12.4 (9.3, 15.5)	129.2
High	25.6 (22.8, 28.3)	40.1 (33.0, 47.2)	14.5 (9.9, 19.2)	56.6
**Diabetes**				
No	18.0 (16.5, 19.4)	32.4 (27.0, 37.8)	14.4 (10.3, 18.6)	80
Yes	47.7 (45.2, 50.2)	59.8 (54.6, 65.0)	12.1 (9.0, 15.2)	25.4
**Dyslipidemia**				
No	17.5 (16.0, 19.0)	31.2 (26.0, 36.5)	13.7 (9.7, 17.7)	78.3
Yes	31.0 (28.8, 33.2)	45.8 (40.7, 51.0)	14.8 (10.9, 18.8)	47.7
**CVD history**				
No	18.7 (17.0, 20.5)	33.5 (27.8, 39.1)	14.7 (10.6, 18.9)	79.1
Yes	62.6 (59.5, 65.5)	69.8 (65.8, 73.9)	7.2 (5.2, 9.2)	11.5
**CKD**				
No	19.2 (17.2, 21.2)	34.1 (28.0, 40.1)	14.9 (10.6, 19.1)	11.5
Yes	37.1 (33.3, 40.8)	47.7 (42.5, 52.9)	10.6 (8.0, 13.2)	28.6
**High ASCVD risk**				
No	18.0 (16.6, 19.5)^a^	32.1 (26.9, 37.4)^b^	14.1 (9.9, 18.4)^c^	78.3
Yes	61.6 (59.1, 64.2)^a^	75.6 (71.6, 79.7)^b^	14.0 (11.9, 16.1)^c^	22.7

**Table 2:** Weighted prevalence and absolute change in hypertension according to JNC7 and ACC/AHA guidelines.

	JNC 7% (95% CI)	ACC/AHA % (95% CI)	Absolute difference
**Males**			
Normal	58.3 (50.9–65.4)	58.3 (50.9–65.4)	0
Prehypertension or elevated blood pressure	22.8 (17.6–29.1)	5.6 (4.1–7.5)	− 17.2
Stage 1 hypertension	16.8 (15.4–18.3)	27.1 (23.3–31.2)	10.3
Stage 2 hypertension	2.1 (1.5–2.9)	9.0 (6.7–12.0)	6.9
**Females**			
Normal	59.2 (54.0–64.2)	59.2 (54.0–64.2)	0
Prehypertension or elevated blood pressure	14.9 (11.3–19.3)	4.0 (2.9–5.5)	− 10.9
Stage 1 hypertension	24.3 (22.9–25.6)	28.7 (26.1–31.5)	4.4
Stage 2 hypertension	1.7 (1.2–2.3)	8.1 (6.2–10.6)	6.4
**Both sexes**			
Normal	58.8 (52.6–64.7)	58.8 (52.6–64.7)	0
Prehypertension or elevated blood pressure	18.9 (14.6–24.2)	4.8 (3.5–6.5)	− 14.1
Stage 1 hypertension	20.5 (19.2–21.7)	27.9 (24.8–31.1)	7.4
Stage 2 hypertension	1.9 (1.3–2.6)	8.6 (6.5–11.2)	6.7

The proportion of awareness among hypertensive adults was 77.5% (73.3, 81.8) based on the JNC7 and 48.6% (41.9, 55.4) based on the ACC/AHA guideline. Among the reclassified participants, awareness was just 2.9% (1.5, 4.3). The reclassified participants aware of their elevated blood pressure compared to participants aware of their traditionally defined hypertension were mostly male (46.8 vs. 36.2%) and were less than 55 years old (64.9 vs. 42.9%), but the proportion of literacy was not different between the two groups. Awareness was higher among females and increased by age based on the ACC/AHA guideline (Fig. 2).

The proportion of treatment among hypertensive adults was 82.2% (77.6, 86.9) and 50.4% (43.3, 57.5) based on the JNC7 and the ACC/AHA guidelines respectively. None of the re-classified participants were treated (Fig. 1). Treatment also increased by age and was higher in females based on both guidelines (Fig. 2).

The proportion of control among hypertensive adults was 63.7% (55.7, 71.7) and 23.3% (14.6, 32.0) based on the JNC7 and the ACC/AHA guidelines respectively. Control among hypertensive adults was higher in females.

The proportion of control among treated was 75.9% (70.2, 81.6) and 46.3% (35.3, 57.4) based on JNC7 and ACC/AHA respectively (Fig. 1). Control among treated participants decreased along with increase in age based on the ACC/AHA guideline.

Finally, based on the JNC7 report, out of the untreated hypertensive adults, a total of 7242 participants (96.9% [95.7, 98.0]) were eligible for pharmacologic treatment and based on the ACC/AHA guideline, 15,258 participants (46.1% [41.6, 50.6]) were eligible. Out of the reclassified participants, 7671 participants (30.7% [25.9, 35.4]) were eligible for pharmacologic treatment (Fig. 1). In short, among the entire study population, 14.1% were newly diagnosed with hypertension based on the ACC/AHA guideline, while only 30.7% of these newly diagnosed adults (4.3% of the entire population) were eligible for pharmacologic treatment.

## Discussion

In the current study, representing a large number of the Iranian population, a total of 6.5 million and 10.7 million Iranians aged 35–70 years, have hypertension based on the JNC7 and the ACC/AHA guidelines, respectively. Although there was some heterogeneity in prevalence across study centers, the application of the ACC/AHA guideline uniformly led to increase in relative prevalence (by 63.7%) and decrease in relative awareness (37.3%), treatment (38.7%), control among hypertensive adults (63.4%), and control among treated adults (39.5%). Yet, the increase in prevalence observed in our study was still lower than previous studies in Iran, which reported a more than a twofold higher prevalence based on the ACC/AHA guideline^16–18^. Less than twofold increases were also observed in other countries^10,11,19,20^.

The results of our study showed that a total of 24,312 adults who were previously classified in the category of “pre-hypertension”, were shifted to stage 1 of hypertension based on the ACC/AHA 2017 guideline. These adults were mainly young and educated males, and many of them free from other metabolic risk factors and comorbidities of high blood pressure with a low 10-year risk of CVD events. These findings may mean that apparently healthy young low-risk male adults may be prone to developing high blood pressure later in life and they shall be detected and managed at early stages, particularly considering the fact that the risk of CVD mortality in males is higher than females, specifically in younger age groups^5,21^.

A similar study in Italy showed that the new blood pressure classification moved 37% of individuals from "pre-hypertension" to "stage 1" and 41% from "stage 1" to "stage 2" hypertension. These results were quite similar to the results of the current study and show that redistribution of hypertensive patients according to the ACC/AHA guideline may help to better identify uncontrolled hypertensive patients with high CVD risk profile^22^.

Implementation of the guideline necessitates that the public be informed and health care professionals use the updated guideline in practice. The result will be a higher number of adults diagnosed with hypertension, who should refer to health care professionals and be managed. There will thus be an apparent additional burden on health care systems. It is worth noting, however, that not all newly-diagnosed adults will require pharmacological treatment. Based on the new guideline, less than one third of the newly diagnosed adults, and mostly elderly groups, will require pharmacological treatment. Therefore, the guidance will not increase medication utilization among the majority, but will hopefully improve awareness and subsequent lifestyle modification before developing very high levels of blood pressure and its accompanying comorbidities later in their lives^13^. The Heart Outcomes Prevention Evaluation (HOPE)-3 trial demonstrated that treatment of adults with intermediate CVD risk has no benefit^23^. Meanwhile, there is recent evidence on cost-effectiveness of a low-cost community-based plan focused on non-pharmacologic but including pharmacologic intervention in three low-income countries (Bangladesh, India, and Sri Lanka)^24^. These results highlight the importance of an integrated non-pharmacological intervention among low and intermediate-risk adults, as recommended in the 2017 ACC/AHA guideline, specifically feasible in low and middle-income countries. Reclassification of adults to higher stages of hypertension compared to previous guidelines is predominantly aimed at improving non-pharmacological interventions and life-style changes. This is the main reason for abolishment of the definition of “pre-hypertension” in recent US guidelines.

The new definition by ACC/AHA was derived from observational studies and clinical trials, focused specifically on results of Systolic Blood Pressure Intervention Trial (SPRINT)^8,25^. There are, however, a number of other studies and trials that don’t support the new criteria and conclude that there is no additional benefit in implementing stricter definitions for hypertension^26,27^. Additionally, although not all newly-labeled hypertensive adults will require pharmacological treatment, there will be an increase in clinical encounters imposing burden of health system infrastructure. According to the AHA/ACC guideline, antihypertensive pharmacologic treatment is initiated for hypertensives with a blood pressure equal or higher than 140/90 mmHg, unless they are high risk^28^. This approach is similar to the ESC/ESH guidelines where for the overwhelming majority of hypertensives, treatment is initiated at this threshold and some consideration for pharmacological treatment can be given to adults with blood pressure 135–139/85–89 mmHg if they report CVD^29–31^. On the other hand, the American College of Physicians and the American Academy of Family Physician guideline was developed for adults 60 years and older and recommended pharmacological treatment to be initiated when SBP was 150 mmHg or higher, unless there was a prior history of stroke or transient ischemic attack, in which case pharmacologic treatment is initiated for blood pressures equal or higher than 140/90 mmHg^32,33^. In actuality, there are more similarities between the guidelines than differences^34^ with the primary difference focused on the definition of stage 1 hypertension. The debate will remain unresolved until longitudinal large-scale studies are conducted on cost-effectiveness and adverse events of different approaches and guidelines^35–37^.

Ultimately, it is worth mentioning that the apparent decrease in awareness, treatment, and control based on the ACC/AHA guideline is due to the fact that neither physicians and health care professionals are aware and use the new guideline in practice, nor the public are informed of the new criteria. Comparing awareness, treatment, and control between various guidelines will only be possible upon their implementation at large scale and for long time periods.

Educational campaigns provide excellent opportunities for improving the awareness of the public and the healthcare workers. World Hypertension Day, promoted by the International Society of Hypertension^38^, and the World Kidney Day^39^ are two exemplar educational campaigns that share this specific goal by providing free blood pressure measurements. In the meantime, using smart phones or tablets can be an excellent option for improving awareness in the general public during the campaigns^40^.

Our study has certain limitations. Despite the large scale of the study and the unique and standard protocol used in its design and implementation, there were variations in outcomes between centers. Therefore, we used study centers as the primary sampling units in our survey data analysis. The cross-sectional design of the study is another limitation that makes it impossible to explore and prove causal relationships. The next limitation is the exclusion of adults younger than 35 years from the study (based on the predetermined protocol of PERSIAN). The fourth limitation is that part of the data in this study was collected before the release of ACC/AHA guideline in 2017. Hereby, we actually aimed to explore what would be the impact if this guideline was applied. And the last limitation of this study is that the mean of first and second blood pressure measurement was used based on JNC7 instead of recording the highest based on the ACC/AHA guideline.

## Conclusions

Overall, our results showed that implementation of the 2017 ACC/AHA guideline will lead to shifting a group of mainly young male adults to the category of stage 1 of hypertension. Future longitudinal studies are mandatory to explore whether the implementation of this strict guideline is cost-beneficial in various settings, especially in low and middle income countries with limited resources. The results of this study demonstrated the “clustering” of metabolic risk factors, which necessitates an integrated approach towards primordial prevention of these risk factors.

## Methods

### Study design

The current study used data from the Prospective Epidemiologic Research Studies in IrAN (PERSIAN) cohort with a population-based cross-sectional design in the baseline recruitment phase. Detailed methods of PERSIAN are published elsewhere^41,42^ In short, a total of 163,770 participants aged 35–70 years were recruited in 18 cohort centers located in 16 provinces in Iran between 2014 and 2020. Participants were recruited through cluster random sampling. The sample was selected to include all ethnic groups in Iran residing in regions with various climates. The exclusion criteria were unwillingness to participate in the study, living in the designated area for less than 9 months, and physical and psychosocial disability impeding the enrollment process. Data collected during the entire 6-year period has been aggregated in the current analysis.

In the first step, trained personnel visited households to invite eligible individuals (based on inclusion criteria) to participate in the study. If individuals agreed to participate, they were requested to refer to their local cohort center in overnight fasting state and to bring the medications they use. Upon arrival, written informed consent form was signed by all participants. They underwent biospecimen collection (blood, urine, hairs, and nails) as well as anthropometric measurements, following protocols established by the US National Institutes of Health^43^. A structured questionnaire including 482 items was filled out during a face-to-face interview. Demographic characteristics, socioeconomic status, lifestyle, past medical history and family history, and medication history were queried. PERSIAN was approved by the ethics committees of the Digestive Disease Research Institute in Tehran University of Medical Sciences and Health Services, and the Medical Sciences Universities supervising each cohort in local study centers. All methods were carried out in accordance with relevant guidelines and regulations. Participants in PERSIAN will be followed for up to 15 years.

### Definitions of outcomes

The main outcomes in this study were prevalence, awareness, treatment, and control of hypertension, and eligibility for treatment based on both JNC7^2,3^ and the 2017 ACC/AHA guidelines^1^. Treatment was defined as self-reported intake or the antihypertensive medications that the participant brought with himself/herself to the study center. Awareness was defined as self-reported history of being diagnosed with hypertension by a physician or a health care professional.

Protocols for blood pressure measurement were developed and validated in the pilot phase of the study. Personnel were meticulously trained by the core team of the PERSIAN to use Riester Exacta 1350 sphygmomanometers across all study centers. Sphygmomanometers were calibrated annually. Trained personnel measured blood pressure in sitting position after 10 min of rest, twice from the right arm and twice from the left arm, with one-minute interval between each of the two consecutive measurements. Personnel were specifically trained to round the measured blood pressure to the nearest 2 mmHg. The average of the second measurements from right and left arms were calculated and considered as the level of blood pressure. Multiple cuff sizes were available for use to best fit the participant’s arm. Trained supervisors at study centers monitored the process of blood pressure measurement and controlled the quality of measurement and data entry. Supervisors used a checklist for monitoring and evaluation of the blood pressure measurement conducted by each of the personnel. Personnel were retrained in case supervisors observed mistakes.

### Definitions of determinants

Demographic characteristics included sex, age, area of residence (rural, urban), and marital status (married versus non-married). Socio-economic status was defined based on education and wealth index. Education was defined in 5 levels: no schooling (< 1 year of primary school), primary school (1–5 years), middle school (6–8 years), high school (9–12 years), and university (> 12 years). Wealth index was calculated using multiple correspondence analysis (MCA) on household assets and divided into 5 quintiles. For physical activity, metabolic equivalents of tasks (METs) were calculated and divided into tertiles. Body mass index (BMI) was calculated and divided into four groups: underweight (< 18.5 kg/m^2^), normal (≥ 18.5 and < 25 kg/m^2^), overweight (≥ 25 and < 30 kg/m^2^), and obese (≥ 30 kg/m^2^). A high waist to hip ratio (WHR) was defined as a ratio ≥ 0.9 in males or ≥ 0.85 in females. Diabetes was defined as self-reported usage of relevant medications or fasting blood sugar (FBS) ≥ 126 mg/dL. Dyslipidemia was defined as low density cholesterol (mg/dL) ≥ 160 and/or total cholesterol (mg/dl) ≥ 240 and/or high density cholesterol (mg/dL) < 40 and/or reporting a history of using lipid lowering medications. Chronic kidney disease (CKD) was defined as glomerular filtration rate (GFR) < 60 ml/min. The 10-year risk of atherosclerosis CVD (ASCVD) based on the ACC/AHA guideline was calculated for all participants^44^.

### Statistical analyses

We calculated the sex and age standardized prevalence of hypertension, the proportion of awareness, treatment, and control among hypertensive patients, the proportion of control among treated patients, and the proportion of untreated adults who were eligible for pharmacologic intervention based on both guidelines. Given the cluster sampling, we used a complex survey design to obtain summary measures. We used sampling weights defined as the inverse probability of being selected in the survey based on data of the national census in 2016. For all estimates, 95% confidence intervals were reported. Data were analyzed using Stata software (version 14.1) (Stata Corp, College Station, TX, USA).

### Ethics approval

PERSIAN was approved by the ethics committees of the Digestive Disease Research Institute in Tehran University of Medical Sciences and Health Services, and the Medical Sciences Universities supervising each cohort in local study centers.

## Data Availability

The data underlying this article will be shared on reasonable request to the corresponding author.

## Abbreviations

ACC: American College of Cardiology
AHA: American Heart Association
ASCVD: Atherosclerotic cardiovascular disease
BMI: Body mass index
CI: Confidence interval
CKD: Chronic kidney disease
CVD: Cardiovascular disease
DBP: Diastolic blood pressure
FBS: Fasting blood sugar
HDL: High-density lipoprotein
JNC7: Seventh report of the Joint National Committee
LDL: Low-density lipoprotein
MCA: Multiple correspondence analysis
MET: Metabolic equivalent of task
PERSIAN: Prospective epidemiologic research studies in IrAN
SBP: Systolic blood pressure
WHR: Waist-to-hip ratio
